# The Association between Histamine 2 Receptor Antagonist Use and *Clostridium difficile* Infection: A Systematic Review and Meta-analysis

**DOI:** 10.1371/journal.pone.0056498

**Published:** 2013-03-04

**Authors:** Imad M. Tleyjeh, Aref A. Bin. Abdulhak, Muhammad Riaz, Musa A. Garbati, Mohamad Al-Tannir, Faisal A. Alasmari, Mushabab AlGhamdi, Abdur Rahman Khan, Patricia J. Erwin, Alex J. Sutton, Larry M. Baddour

**Affiliations:** 1 Department of Medicine, King Fahad Medical City, Riyadh, Saudi Arabia; 2 Division of Infectious Diseases, Mayo Clinic, S.W, Rochester, Minnesota, United States of America; 3 Division of Epidemiology, Mayo Clinic, S.W, Rochester, Minnesota, United States of America; 4 Department of Internal Medicine, School of Medicine, University of Missouri – Kansas City, Kansas City, Missouri, United States of America; 5 Research and Scientific Publication Center, King Fahad Medical City, Riyadh, Riyadh, Saudi Arabia; 6 Department of Internal Medicine, University of Toledo Medical Center, Toledo, Ohio, United States of America; 7 Mayo Medical Library, Mayo Clinic, S.W, Rochester, Minnesota, United States of America; 8 Department of Health Sciences, University of Leicester, Leicester, England; Universidad Peruana de Ciencias Aplicadas (UPC), Peru

## Abstract

**Background:**

*Clostridium difficile* infection (CDI) is a major health problem. Epidemiological evidence suggests that there is an association between acid suppression therapy and development of CDI.

**Purpose:**

We sought to systematically review the literature that examined the association between histamine 2 receptor antagonists (H_2_RAs) and CDI.

**Data source:**

We searched Medline, Current Contents, Embase, ISI Web of Science and Elsevier Scopus from 1990 to 2012 for all analytical studies that examined the association between H_2_RAs and CDI.

**Study selection:**

Two authors independently reviewed the studies for eligibility.

**Data extraction:**

Data about studies characteristics, adjusted effect estimates and quality were extracted.

**Data synthesis:**

Thirty-five observations from 33 eligible studies that included 201834 participants were analyzed. Studies were performed in 6 countries and nine of them were multicenter. Most studies did not specify the type or duration of H_2_RAs therapy. The pooled effect estimate was 1.44, 95% CI (1.22–1.7), I^2^ = 70.5%. This association was consistent across different subgroups (by study design and country) and there was no evidence of publication bias. The pooled effect estimate for high quality studies was 1.39 (1.15–1.68), I2 = 72.3%. Meta-regression analysis of 10 study-level variables did not identify sources of heterogeneity. In a speculative analysis, the number needed to harm (NNH) with H_2_RAs at 14 days after hospital admission in patients receiving antibiotics or not was 58, 95% CI (37, 115) and 425, 95% CI (267, 848), respectively. For the general population, the NNH at 1 year was 4549, 95% CI (2860, 9097).

**Conclusion:**

In this rigorous systematic review and meta-analysis, we observed an association between H_2_RAs and CDI. The absolute risk of CDI associated with H_2_RAs is highest in hospitalized patients receiving antibiotics.

## Introduction

*Clostridium difficile* infection (CDI) is considered a major health problem with a point prevalence of 13.1/1000 in-patient [1] and is increasing in incidence and mortality [2]–[5]. The CDI cost in the United States of America (USA) alone was conservatively estimated to exceed $1.1 billion annually [6]. Risk factors associated with CDI acquisition are numerous and traditionally have included exposure to antibiotics, advanced age, comorbidities, enteral feeding, prolonged hospitalization, endoscopy and antineoplastic medications [7]–[10].

The role of gastric acid suppression therapy has gained interest recently as a risk factor for CDI. Four recently published meta-analyses have suggested an association between gastric acid suppression therapy with proton pump inhibitors (PPI) and CDI [11]–[14]. The United States Food and Drug Administration (FDA) recently warned the public about a possible association between CDI and PPI use [15]. However, to date; there is no systematic review dedicated to evaluate the potential association between histamine _2_ receptors antagonists (H_2_RAs) use and risk of CDI.

H_2_RAs are popular over-the-counter (OTC) drugs worldwide [16]. Off -label use of H_2_RAs and substitution for physician care were reported in 46 % and 34% of the adult consumer, respectively [15]. Masking serious conditions, missed diagnosis, and the potential for inappropriate use by patients are concerns about OTC use of H_2_RAs [17]. Nonetheless, the implications of OTC H_2_RAs use are not yet well defined.

Given the high prevalence of prescription use and OTC use of H_2_RAs and the increasing incidence and severity of CDI, we sought to systematically review the published literature that examined the association between H_2_RAs use and development of CDI following the MOOSE [18] and PRISMA [19] guidelines. We use the Grades of Recommendation, Assessment, Development and Evaluation (GRADE) framework [20] to interpret our findings.

## Methods

### Search strategy

The search strategy and subsequent literature searches were performed by a medical reference librarian (PJE) with 37 years of experience. The initial strategy was developed in Ovid MEDLINE (1990 through January 2012), using MeSH (Medical Subject Headings) controlled vocabulary, and then modified for Ovid EMBASE (1990 through January 2012). Primary terms were: enterocolitis, pseudomembranous/ AND the therapeutic agents of interest: explode omeprazole, explode proton pump inhibitors, anti-ulcer agents, and explode histamine H_2_ antagonists (Explode allows including all of the specific drugs, without having to use all of the various terms, synonyms, brands and generic names.) Articles were limited to randomized controlled trials, cohort studies, and or case-control studies. The same process was used with Ovid EMBASE with alterations as necessary to accommodate EMBASE's more granular subject headings. ISI Web of Science and Elsevier Scopus use text words: (difficile OR pseudomembranous OR pseudo-membranous) AND (omeprazole OR “proton pump” OR ranitidine OR h2 OR h-2 OR “acid suppression” OR antacid*)) AND (random* OR trial* OR blind* OR cohort* OR controlled OR prospective). Moreover, bibliographic references of all articles and previous meta-analyses were searched for eligible studies. We have designed the search strategy to capture any association between gastric acid suppression therapy and development of CDI.

There was no restriction to language. All results were downloaded into EndNote 7.0 (Thompson ISI Research soft, Philadelphia, Pennsylvania), a bibliographic database manager, and duplicate citations were identified and removed. Two authors (A.B.A. and F.A.) independently assessed the eligibility of identified studies.

### Study selection

To be included, a study had to: (1) be an analytical study; and (2) examine the association between H_2_RAs use and incidence of CDI in adult population.

### Data collection

A data collection form was developed and used to retrieve information on relevant features and results of pertinent studies. Two reviewers (A.B.A. and F.A.) independently extracted and recorded data in a predefined checklist. Disagreements among reviewers were discussed with two other reviewers (I.M.T. and M.A.A.), and agreement was reached by consensus. We collected adjusted effect estimates and 95% confidence intervals (CI) based on the multivariable regression model used in each study.

We used the Newcastle-Ottawa Quality Assessment Scale for cohort and case-control studies [21] which is intended to rate selection bias, comparability of the exposed and unexposed groups of each cohort, outcome assessment, and attrition bias. Two reviewers (M.A.G and F.A.) independently assessed the methodological quality of selected. Disagreement among reviewers was discussed with 2 other reviewers (I.M.T. and M.A.A.), and agreement was reached by consensus.

We used the GRADE framework to interpret our findings. The Cochrane Collaboration has adopted the principles of the GRADE system [20] for evaluating the quality of evidence for outcomes reported in systematic reviews.

For purposes of systematic reviews, the GRADE approach defines the quality of a body of evidence as the extent to which one can be confident that an estimate of effect or association is close to the quantity of specific interest. Quality of a body of evidence involves consideration of within-study risk of bias (methodological quality), directness of evidence, heterogeneity, precision of effect estimates and risk of publication bias.

### Statistical Analyses

#### Meta-analyses

The primary effect measures used in the meta-analysis were Odds Ratios (OR), Hazard Ratios (HR) and Relative Risks (RR) which were assumed to reasonably estimate the same association between CDI and H_2_RAs given the low incidence of CDI and thus were pooled together. Adjusted effect estimates were primarily used for this analysis. Unadjusted effect estimates were used as alternatives if studies did not pursue adjustment because of absence of association on univariate comparison.

Effect estimates from all included studies were pooled in a meta-analysis weighing individual studies according to their log-transformed inverse variance. The DerSimonian and Laird random effects model [22] was used to calculate the pooled effect estimates.

We extracted data on the proportion of CDI cases that were exposed to antibiotics from all studies that reported these data. We then performed a meta-analysis for the proportion on logit scale using random effects model weighing the individual studies according to their log-transformed inverse variance.

#### Exploring heterogeneity

Homogeneity among studies was tested by means of Cochran's Q test and calculation of the variation across studies attributable to heterogeneity rather than chance (I^2^). The influence of a range of a-priori selected study-level and aggregated individual-level parameters on the observed effect estimate was investigated by means of meta-regressions. In these analyses, the log odds ratio from each study was regressed on the potential confounders in univariate and multivariate weighted linear regressions, weighted according to the inverse standard error and the residual between-study variance. Ten potential confounders were considered. Seven variables were categorical: design of the study (case-control vs. cohort), country of publication, setting (single center vs. multicenter), method of ascertainment of antibiotic use, method of effect measure (OR vs. RR/HR), effect estimate (adjusted vs. unadjusted) and quality of included studies (high score vs. low score). Three continuous variables were: the impact factor of the journal where the study was published, number of variables the effect measure was adjusted for and proportion of cases that were exposed to antibiotics.

#### Publication bias

The possible influence of publication bias was graphically assessed with the novel method of contour-enhanced funnel plot where log-transformed odds ratios were plotted against standard errors. This method examines whether any funnel plot asymmetry is likely to be due to publication bias compared with other underlying causes of funnel plot asymmetry. The contours help to indicate whether areas of the plot, where studies are perceived to be missing, are where studies would have statistically significant effect sizes or not and thus decrease or increase the evidence that the asymmetry is due to publication bias. The presence of funnel plot asymmetry was also assessed using Egger's test [23].

#### Residual confounding

Finally, the possible influence of unknown confounders (residual confounding) was investigated with a rule-out approach described by Schneeweiss [24]. This approach stipulates the influence of a hypothetical confounder and determines what characteristics this confounder must have to fully account for the observed association between use of H_2_RAs and occurrence of CDI. The hypothetical confounder is characterized by its association to H_2_RAs use (OR_EC_, odds ratio of exposure to the confounder) and its association to the outcome (RR_CO_, relative risk of outcome in individuals exposed to the confounder vs. non-exposed). For this analysis, the absolute risk in the pooled non-exposed group was used for conversion of odds ratio to relative risk using the method described by Zhang and Yu [25]. Separate analyses were performed to demonstrate what levels of OR_EC_ and RR_CO_ would be required to fully explain the observed association between H_2_RAs and CDI for different hypothetical prevalence of the unknown confounder (i.e. P_C_ = 0.2, P_C_ = 0.4) before and after adjustment for publication bias as described above.

In all analyses, results associated with p-values <0.05 (two-sided test) were considered statistically significant. All statistical analyses were performed using Stata version 12 statistical software (StataCorp, College Station, Texas).

## Results

### Search results

The search yielded 27 eligible studies after excluding 260 citations. Six more studies were retrieved from recent review articles and added to the total eligible studies. Kutty [26] et al and Jayatilaka [27] et al, each reported 2 different observations for different participants. Thus, a total of 33 articles met our inclusion criteria representing 35 observations that included 201834 participants. There was excellent agreement for the inclusion of the studies, data abstraction and quality assessment between the reviewers (kappa statistic being 1.0, 1.0 and 0.91 respectively).

**Figure 1:**
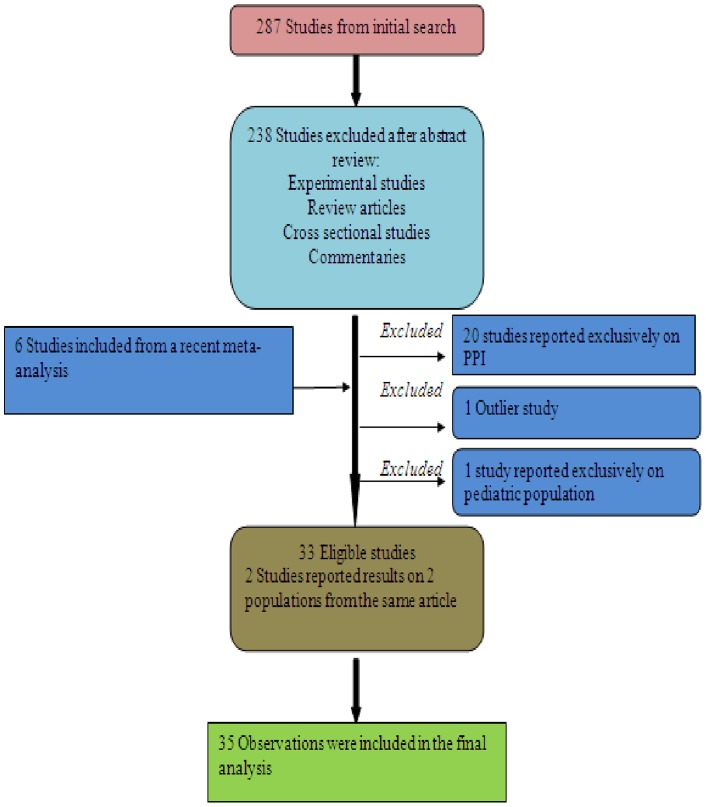
Flow diagram of eligible studies.

The study selection process is illustrated in Figure 1 and the main characteristics of the included studies are summarized in Table 1. Twenty-four case control studies [26]–[37], [45], [49]–[51], [53]–[58] and 11 cohort studies [38]–[44], [46]–[48], [52] reported data on both community-acquired and hospital-acquired CDI (8 observations were from community-acquired CDI, 23 from hospital-acquired CDI and 4 representing both type of CDI). Six studies [26], [39], [47], [51], [53], [54] were from multiple centers; two from UK general practice research database [28], [30], and the remaining were from single centers. The included studies were performed in 6 countries (17 studies from USA, 9 from Canada, 6 from United Kingdom, 1 from Netherlands, 1 from Israel, and one from Korea). Most studies did not specify the type or duration of therapy with H_2_RAs. Tables 2 and 3 summarized the case ascertainment, control or non-exposed group selection method for case control and cohort studies, respectively. Among all citations, seventeen studies reported the proportion of cases exposed to antibiotics. Eight studies used antibiotics exposure as inclusion criteria. Three studies did not provide either the absolute number of exposed or unexposed groups thus were not included in this pooled proportion analysis.

**Table 1:** Characteristics of the included studies.

Source	Country	Centers	Setting	Condition	Study Design	Inclusion Criteria	Acid Suppression Therapy
**Kutty et al (VA),^26^2010**	US	Multicenter	Community	Gen Pop	Case-control	Age: ≥18 yr; Community****onset CDAD	**H_2_RAs: exposure 3mo prior to test**
**Kutty et al (D),^26^ 2010**	US	Multicenter	Community	Gen Pop	Case-control	Age: ≥18 yr; Community****onset CDAD	**H_2_RAs: exposure 3mo prior to test**
**Nath et al, ^28^1994**	CA	Single	Hospital	Hem-onco pts	Case-control	Adult; In-patient >3d	**Acid suppression therapy**
**Jayatilaka et al, ^27^ 2007**	US	Single	Hospital	Gen In-patient	Case-control	Age >18	**H_2_RAs: pre admission**
**Jayatilaka et al,^27^2007**	US	Single	Hospital	Gen In-patient	Case-control	Age >18	**H_2_RAs: post admission**
**Shah et al (D),^29^ 2000**	UK	Single	Hospital	Gen In-patient	Case-control	Age >65 yr; Gen medical/elderly****care wards	**H_2_RAs: upto 16 wk before diarrhea**
**Dial et al, ^30^ 2005**	UK	GPRD	Community	Gen Pop	Case-control	Age ≥18 yr; At least 2 yrs of records****in the GPR; first****occurrence of CDAD	**H_2_RAs: 90 d prior to the index date**
**Debast et al, ^31^ 2009**	NL	Single	Hospital	Gen In-patient	Case control	Age:≥18 yr; CDAD	**H_2_RAs: exposure**
**Lowe et al, ^32^ 2006**	CA	Single	Community	Gen Pop	Case-control (R)	1 hospital admission for CDAD; Age ≥ 66yr; CDAD diagnosis within 60d of ABX therapy	**H_2_RAs: exposure**
**Dial et al, ^33^ 2006**	UK	GPRD	Community	General pop	Case-control	First prescription oral Vancomycin; No previous admission 1yr before index date	**H_2_RAs: 90d prior to index date**
**Aseeri et al, ^34^ 2008**	US	Single	Hospital	Gen In-patient	Case-control	Age ≥18 Yr; Inpt for ≥3 d	**H_2_RAs: 3d before CDAD**
**Dubberke et al,^35^ 2007**	US	Single	Hospital	Gen In-patient	Case- Control	Pts admitted for >48 hr****between study period	**H_2_RAs**
**Loo et al, ^36^ 2005**	UK	Single	Hospital	Gen In-patient	Case-control	Hospital Acquired CDAD;	**H_2_RAs: 6wk before diagnosis**
**Sundram et al,^37^ 2009**	UK	Single	Hospital	Gen In-patient	Case-control	Adult Hospital****Acquired CDAD	**H_2_RAs: 6wk prior to onset**
**Howell et al,^38^ 2010**	US	Single	Hospital	Gen In-patient	Cohort	Age ≥18 yr; LOS ≥3 d;****Only first diagnosis	**H_2_RAs**
**Dalton et al, ^39^ 2009**	CA	Multicenter	Hospital	Med/Surgical Subspecialty	Cohort, (R)	Age: ≥18 yr; Minimum 7-d****LOS; ABX exposure	**H_2_RAs**
**Dubberk et al,^40^2007**	US	Single	Hospital	Gen In-patient	Cohort, (R)	All pts admitted to BJH****for more than 48 hours	**H_2_RAs**
**Pepin et al,^41^ 2005**	CA	Single	Hospital	Gen In-patient	Cohort, (R)	Adult In-patient	**H_2_RAs**
**Beaulieu et al, ^42^ 2007**	CA	Single	Hospital	Medical ICU	Cohort	ICU LOS>24hr; Diarrhea >24 hr and positive CD toxin (2d to 2months****post discharge)	**H_2_RAs**
**Peled et al, ^43^ 2007**	IL	Single	Hospital	Gen In-patient	Cohort, (P)	CD testing during 4m period; ABX within 40d prior to diarrhea	**H_2_RAs**
**Dial et al, ^44^ 2004**	CA	Single	Hospital	Med/CT/Surgical wards	Cohort	Pharmacy database; ABX during study****period; positive toxin in the****infection control registry	**H_2_RAs**
**Novell et al, ^45^, 2010**	US	Single	Hospital	Gen Inpatients	Case-control, (R)	Age ≥18 yr; CDAD	**H_2_RAs**
**Netland et al, ^46^ 2011**	US	Single	Both	Gen Pop	Cohort, (R)	Recurrent CDI	**H_2_RAs**
**Jung et al, ^47^ 2010**	Korea	Single	Hospital	Gen Inpatients	Cohort study, (R)	Recurrent CDAD or****treatment failure cases	**H_2_RAs**
**Loo et al, ^48^ 2011**	CA	Multicenter	Hospital	Gen Inpatients	Cohort study(P)	Age ≥18, Health Care****Associated CDAD	**H_2_RAs**
**Manges et al, ^49^ 2010**	CA	Single	Hospital	Gen Inpatients	Case control	Nosocomial CDAD	**H_2_RAs**
**Kuntz et a,l^50^ 2011**	US	Single	Community	Gen Pop	Case control, (R)	Community****Associated CDAD	**Acid suppression therapy**
**Naggie et al,^51^ 2011**	US	Multicenter	Community	Gen Pop	Case control	Age≥18 yr	**Acid suppression therapy**
**Stevens et al,^52^, 2011**	US	Single	Hospital	Gen Inpatients	Cohort, (R)	Age ≥18 yr,****Hospital acquired	**H_2_RAs**
**Dial et al, ^53^ 2008**	CA	Multicenter	Community	Elderly patients	Case control	Age ≥65, Community****Associated CDAD	**H_2_RAs**
**McFarland et al,^54^ 2007**	US	Multicenter	Both	Gen Pop	Case control	CDAD Diagnosis	**H_2_RAs**
**Kazakova et al,^55^ 2012**	US	Single	Both	Gen Pop	Case control	CDAD Diagnosis, onset during****the pre-outbreak or outbreak****periods, hospitalization	**H_2_RAs**
**Modena et al,^56^ 2005**	US	Single	Both	Gen Pop	Case control	Received at least 5 days****of antibiotics prior to diagnosis of CDAD	**H_2_RAs**
**Muto et al,^57^ 2005**	US	Single	Hospital	Gen Inpatients	Case control	Nosocomial CDAD	**H_2_RAs: During the 4 weeks before detection of CDAD**
**Yip et al, ^58^ 2001**	**CA**	**Single**	**Hospital**	**Gen Inpatients**	**Case control**	**Nosocomial CDAD**	**H_2_RAs**

Abbreviations: US, United States;UK, United Kingdom; BMT, Bone Marrow Transplant; ESRD, End Stage Renal Disease; GPRD – general practice research database; IBD, Inflammatory Bowel Disease; CD, Clostridium Difficile; CDAD, Clostridium difficile associated diarrhea; LOS, Length of Stay; LTCF, Long Term Care Facility; Gen, General.; Pop, Population; d, day/days; mo, month/months; yr, year/year; wk, week/week; Pts, Patients; Pt, Patient; Med, Medical; CT, Cardio-thoracic; NL, Netherland; CA, Canada; IL, Israel; Abd, Abdominal; (P), prospective; (R), Retrospective.*, Mostly hospital.

**Table 2:** The Association between H_2_RAs use and development of *Clostridium difficile* infection from case-control studies.

Source	Case Ascertainment	Selection of Controls	Sample size	Adjusted Effect Estimates
Kutty et al,^26^ (VA)	Non-formed stool,****Positive CD toxin	Randomly selected****from the same geographical****outpatients territory	Exposed group; cases:****7, controls: 13	Crude OR, 1.8 (0.6–4.8)
			Non-exposed group;****cases: 29, controls: 95	
Kutty et al,^26^ (D)	Non-formed stool,****Positive CD toxin	Randomly selected from****the same geographical outpatients territory	Exposed group; cases:****6, controls: 3	Crude OR, 1.3 (0.3–5.6)
			Non-exposed group;****cases: 67, controls: 45	
Sundram et al,^37^ 2009	Diarrhea, Positive stool****for CD toxin, ribotyped	Inpatients, No diarrhea, Never****tested positive for CD	Exposed group; cases:****65, controls: 52	Crude OR for PPI/ H_2_RAs :****1.7, P 0.456
			Non-exposed group;****cases: 32, controls: 45	
Jayatilaka et al,^27^ 2007	Diarrhea, Positive toxin	Age and sex matched,****Same period of time	H_2_RAs use pre and****during admission	H_2_RAs use pre and****during admission
			Exposed group; cases:****9, controls: 17	OR: 0.95****(0.39-2.34)
			Non-exposed group;****cases: 6, controls: 14	
Jayatilaka et al,^27^ 2007	Diarrhea, Positive****toxin	Age and sex matched,****Same period of time	H_2_RAs****use post admission	H_2_RAs****use post admission
			Exposed group; cases:****133, controls: 227	OR: 0.73****(0.26-2.06)
			Non-exposed group;****cases: 116, controls: 230	
Loo et al,^36^ 2005	Diarrhea/positive CD, Endoscopic****diagnosis, histological evidence	Matched to Age,****Charlson index, date of****admission, ward, LOS	Exposed group; cases:****47, controls: 47	Diarrhea/positive CD, Endoscopic****diagnosis, histological evidence
			Non-exposed group;****cases: 190, controls: 190	
Shah et al,^29^ 2000	Diarrhea Positive****stool for CD toxin	Negative stool toxins,****Similar age, Hospital****ward, Same time	Exposed group; cases:****22, controls: 22	Diarrhea
			Non-exposed group;****cases: 104, controls: 104	Positive stool****for CD toxin
Dial et al,^33^ 2006	Patients with first prescription****of oral Vancomycin	Age matched,****Same ward	Exposed group; cases:****23, controls: 112	Patients with first prescription****of oral Vancomycin
			Non-exposed group;****cases: 294, controls: 2055	
Asseri et al,^34^ 2008	Diarrhea	Matched to date of****admission, antibiotic use, gender, age group, patient location, room type	Exposed group; cases:****17, controls: 9	Diarrhea
	Positive stool****for CD toxin		Non-exposed group;****cases: 77, controls: 85	Positive stool****for CD toxin
Dial et al,^30^ 2005	Positive CD toxin	Same general practice,****Not hospitalized in the year****prior to index date, Negative CD toxin, No diagnosis of CDI	Exposed group;****cases: 83, controls: 367	Positive CD toxin
	Clinical diagnosis****made by GP		Non-exposed gp;****cases: 1150, controls: 11963	Clinical diagnosis****made by GP
Lowe et al,^32^ 2006	CDAD	Matched to age, sex,****and antibiotic use	Exposed group; cases: 213, controls: 1846****Non-exposed gp; cases: 1176, controls: 10457	Exposed group; cases: 213, controls: 1846****Non-exposed gp; cases: 1176, controls: 10457
Debast et al,^31^ 2009	Diarrhea	Randomly selected fro****the same time and same wards as****CDI cases	Exposed group; cases:****2, controls: 2	Exposed group; cases:****2, controls: 2
	Positive stool****for CD toxin		Non-exposed group;****cases: 43, controls: 88	Non-exposed group;****cases: 43, controls: 88
Nath et al,^28^ 1994	Diarrhea	Age matched,****Same hospital unit	Exposed group; cases:****51, controls: 32	Exposed group; cases:****51, controls: 32
	Positive stool****for CD toxin		Non-exposed group;****cases:29, controls: 48	Non-exposed group;****cases:29, controls: 48
Dubberke et al,^35^ 2007	Diarrhea	Randomly selected****During the study period	Exposed group; cases:****206, controls: 426	Exposed group; cases:****206, controls: 426
	Positive stool****for CD toxin		Non-exposed group;****cases: 176, controls: 1102	Non-exposed group;****cases: 176, controls: 1102
Novell et al,^45^ 2010	New diarrhea	Matched to in-patient unit,****age, gender, date of admission	Exposed group; cases:****12, controls: 07	Exposed group; cases:****12, controls: 07
	Positive stool****for CD toxin		Non-exposed group;****cases: 162, controls: 167	Non-exposed group;****cases: 162, controls: 167
Manges et al,^49^ 2010	Diarrhea/positive CD, Endoscopic****diagnosis, histological evidence	Matched to Age, gender,****date of hospitalization	Exposed group; cases:****09, controls: 12	Exposed group; cases:****09, controls: 12
			Non-exposed group;****cases: 16, controls: 38	Non-exposed group;****cases: 16, controls: 38
Kuntz et al,^50^ 2011	ICD-9 code, CDAD	Randomly selected	Exposed group; cases:****55, controls: 157	Exposed group; cases:****55, controls: 157
			Non-exposed group;****cases: 249, controls: 2883	Non-exposed group;****cases: 249, controls: 2883
Naggie et al,^51^ 2011	Diarrhea	Matched by geographic****location	Exposed group; cases:****22, controls: 44	Exposed group; cases:****22, controls: 44
	Positive stool****for CD toxin		Non-exposed group;****cases: 44, controls: 70	Non-exposed group;****cases: 44, controls: 70
Dial et al,^53^ 2008	ICD-9 code****008.45, CDAD	Randomly selected, matched****to index date and date of first hospital admission	NR	RR:1.60 (0.90-2.20)
McFarland et al,^54^ 2007	Acute diarrhea Culture positive or positive C.D toxins	Matched to time of****CDAD, Age, Ward	Exposed group; cases:****24, controls: 160	NR
	No other cause****for the diarrhea		Non-exposed group;****cases: 23, controls: 161	
Kazakova et al,^55^ 2012	Diarrhea, positive****CD toxin A	Matched to Sex, Age,****admission date	Exposed group;****cases:19, controls: 49	OR:2.69****(1.22-5.97)
			Non-exposed group;****cases: 18, controls: 109	
Modena et al,^56^ 2005	Diarrhea	Inpatients, Received****antibiotics for at least 5 days	Exposed group;****cases:32, controls:18	NR
	Positive stool****for CD toxins		Non-exposed group;****cases: 98, controls: 102	
Muto et al,^57^ 2005	Diarrhea	Matched to admission date,****Type of medical service, Length of hospital stay	Exposed group;****cases:159, controls:44	OR:2.00****(1.10-3.50)
	Positive stool****for CD toxin		Non-exposed group; cases: 141, controls: 62	
Yip et al,^58^ 2001	Diarrhea	Matched to Age,****Gender, admission date	Exposed group;****cases:14, controls:13	OR:2.70****(0.71–10.10)
	Positive stool****for CD toxin		Non-exposed group;****cases: 9, controls: 18	

**Table 3:** The Association between H2RAs use and development of *Clostridium difficile* infection from cohort studies.

Source	Case Ascertainment	Selection of Controls	Sample size	Adjusted Effect Estimates
**Howell et al,^38^ 2010**	Positive CD toxin	A nearest-neighbor–****matching algorithm****was applied	Exposed group; cases: 66,****controls: 10619	OR : 1.53 (1.12–2.10)
			Non-exposed group; cases:599,****controls: 90512	
**Dalton et al,^39^ 2009**	Positive stool toxins or colonoscopy-****confirmed psudomembraneous colitis	Age, ≥ 18 years, Minimum****7d LOS, Antibiotic exposure	Exposed group; cases:****28 controls: 2135	OR, 1.70 (1.09 2.64)
			Non-exposed group;****cases:121, controls: 12435	
**Dubberk et al,^40^ 2007**	Positive stool for CD	In-patient, No positive****stool toxin assay during the period****(60d before start****of study to the end)	Exposed group; cases: 206,****controls: 998 Non-exposed group;****cases: 176, controls: 25716	OR, 2.0 (1.6-2.6)
**Pepin et al, ^41^ 2005**	Diarrhea, Positive toxin, proven****pseudomembranous colitis	Unclear	Exposed group; cases:****1199, controls: NR	HR, 1.07 (0.8-1.43)
			Non-exposed gp; cases:****6222, controls: NR	
**Beaulieu et al,^42^ 2007**	Diarrhea****Positive stool for CD toxin	Unclear	Exposed group; cases:****470, controls: NR	HR, 0.78 (0.5 – 1.23)
			Non-exposed group;****cases: 357, controls: NR	
**Peled et al,^43^ 2007**	Diarrhea****Positive stool for CD toxin	Diarrhea with negative****stool for CD, same****institution	Exposed group; cases:****22, controls: 45	OR, 3.1 P value : 0.024
			Non-exposed group;****cases: 30, controls: 120	
**Dial et al,^44^ 2004**	Positive stool****for CD toxins	Unclear	Exposed group; cases:****NR, controls: NR	OR : 1.1 (0.4-3.4)
			Non-exposed group;****cases: NR, controls: NR	
**Netland et al, ^46^ 2011**	Diarrhea between 5–60 days****after antibiotic therapy for CDAD	Patients with CDAD in****the same institution	Exposed group; cases:****05, controls: 50	OR, 0.49 P value : 0.33
			Non-exposed group;****cases: 50, controls: 99	
**Jung et al,^47^ 2010**	Diarrhea or pseudomembranous****colitis, Positive toxin	Same institution	Exposed group; cases:****06, controls: 31	OR, 1.59 P value : 0.367
			Non-exposed group;****cases: 08, controls: 66	
**Loo et al,^48^ 2011**	Diarrhea and: positive CD, histological evidence****or pseudomembranous colitis	Frequency matching****approach	Exposed group; cases:****NR, controls: NR	OR : 0.55 (0.21 – 1.49)
			Non-exposed group;****cases: 190, controls: 190	
**Stevens et al,^52^ 2011**	Diarrhea Positive****stool for CD toxin	Same institution	Exposed group; cases:****23, controls: 1060	HR, 1.7 (0.7 – 3.9), P value 0.25
			Non-exposed group;****cases: 218, controls: 8853	

### Quality assessment

Quality assessment of all included studies was done using the validated Newcastle-Ottawa Quality Assessment Scale [21] for cohort and case control studies (Tables 4 and 5). Included studies were scored based on the sum number of the stars given to each study. Among case-control studies, Loo et al 2011, Manges et al 2010, McFarland et al 2007, Modena et al 2005 and Dial et al 2008 scored the lowest. While Beaulieu et al 2005 scored the lowest among cohort studies. Most studies were of good quality with no evidence of selection bias, and with good comparability of the exposed and unexposed groups of each cohort, and outcome assessment.

**Table 4:** Modified Newcastle-Ottawa quality assessment scale for case-control studies included in the meta-analysis.

	Selection*					Exposure^0^			
Included Studies	Adequacy of Case Definition	Representativeness of the Cases	Selection of Controls	Definition of Controls	Comparability•	Ascertainment of Exposure	Same Method of Ascertainment for Cases and Controls	Non- ResponseRate	Total No. of stars
Kutty et al,^26^ 2010.	A*	A*	A*	A*	A*	A*	A*	C	7
Nath et al,^28^1994	A*	A*	B	A*	A**	A*	A*	C	7
Jayatilaka et al,^27^ 2007	B	A*	B	A*	A**	A*	A*	C	6
Shah et al,^29^ 2000	A*	A*	B	A*	A*	A*	A*	C	6
Lowe et al,^32^ 2006	A*	A*	A*	A*	A*	A*	A*	C	7
Dial et al,^30^ 2005	A*	A*	A*	A*	A*	A**	A*	C	8
Dial et al,^33^ 2006	B	A	A*	A	A*	A**	A*	C	5
Aseeri et al,^34^ 2008	A*	A*	B	A*	A**	E	B*	C	6
Dubberke et al,^35^ 2007	A*	B	B	A*	A**	A*	A*	C	6
Loo et al,^36^ 2005	A*	A*	B	A*	A*	E	A*	C	5
Sundram et al,^37^ 2009	A*	A*	B	A*	A*	A*	A*	C	6
Novell et al,^45^ 2010	A*	A*	B	A*	A**	A*	A*	C	7
Debast et al,^31^ 2009	A*	A*	B	A*	A*	A*	A*	C	6
Kuntz et al,^50^ 2011	A*	A*	A*	A*	A*	A*	A*	C	7
Manges et al,^49^ 2010	A*	A*	B	B	A*	A*	A*	C	5
Naggie et al,^51^ 2011	A*	A*	A*	A*	A*	C	A*	C	6
McFarland et al,^54^ 2007	B	A*	C	A*	A*	A*	A*	C	6
Modena et al,^56^ 2005	B	A*	B	A*	A**	A*	A*	C	5
Muto et al,^57^ 2005	B	A*	B	A*	A**	A*	A*	C	6
Yip, et al,^58^ 2001	B	A*	B	A*	A**	A*	A*	C	6
Dial et al,^53^ 2008	B	A*	B	A*	A*	A*	A*	C	5
Kazakova et al,^55^ 2006	A*	A*	B	A*	A**	D	A*	C	6

* Selection:

(1)Is this case definition adequate? A, yes, with independent validation; B, yes, eg record linkage or based on self reports C, no description.

(2) Representativeness of the cases: A, Consecutive or obviously representative series of cases; B, Potential for selection biases or not stated.

(3) Selection of controls: A, Community controls; B, Hospital controls; C, No description.

(4) Definition of controls: A, No history of disease; B, No description of source.

• Comparability: Comparability of cases and controls on the basis of the design or analysis: A, study controls for co-morbidities; B, study controls for any additional factor (e.g., age and severity of illness).

0 Exposure:

Ascertainment of exposure: A, Secured records; B, Structured interview where blind to case/control status; C, Interview not blinded to case/control status; D, written self report or medical record only.

Same method of ascertainment for cases and controls; A, yes; B, no.

Non-response rate: A, Same for both groups; B, Non-respondents described; C, Rate different and no designation.

**Table 5:** Modified Newcastle-Ottawa Quality Assessment Scale for Cohort studies included in the Meta-analysis

	Selection*					Outcome^0^			
Included Studies	Representativeness of the exposed cohort	Selection of the Non-exposed Cohort	Ascertainment of Exposure	Incident Disease	Comparability•	Assessment of Outcome	Length of Follow-up	Adequacy of Follow-up	Total number of stars
Howell et al,2010	A*	A*	A*	A*	A**	B*	A*	A*	9
Dalton et al, 2009	A*	A*	A*	A*	A**	B*	A*	A*	9
Dubberke et al, 2007	A*	A*	A*	A*	A**	B*	A*	A*	9
Pepin et al, 2005	A*	A*	A*	A*	A*	B*	A*	A*	8
Beaulieu et al, 2007	B	A*	A*	A*	A*	A*	A*	A*	7
Peled et al, 2007	A*	A*	B	A*	A**	A*	A*	A*	8
Loo et al 2011	A*	A*	B	A*	A**	A*	A*	A*	8
Netland et al, 2011	A*	A*	A*	A*	A**	B*	A*	A*	9
Jung et al, 2010	A*	A*	A*	A*	A**	B*	A*	A*	9
Stevens et al, 2011	A*	A*	A*	A*	A**	B*	A*	A*	9
Dial et al 2004	A*	A*	A*	A*	A*	A*	A*	A*	8

* Selection:

(1) Representativeness of the exposed cohort: A, truly representative; B, somewhat representative; C, selected group; D, no description of the derivation of the cohort.

(2) Selection of the non-exposed cohort: A, drawn from the same community as the exposed cohort; B, drawn from a different source; C, no description of the derivation of the non-exposed cohort.

(3) Ascertainment of exposure: A, secure record; B, structured interview; C, written self-report; D, no description.

(4) For demonstration that the outcome of interest was not present at start of study: A, yes; B, no.

• Comparability: For comparability of cohorts on the basis of the design or analysis: A, study controls for co-morbidities; B, study controls for any additional factor (e.g., age and severity of illness); C, not done.

0 Outcome:

(1) Assessment of outcome: A, independent blind assessment; B, record linkage; C, self-report; D, no description.

(2) Was follow-up long enough for outcomes to occur? A, yes, (i.e. in-hospital or up to 30 d); B, no.

(3) Adequacy of follow-up of cohorts: A, complete follow-up and all subjects accounted for; B, subjects lost to follow-up was unlikely to introduce bias, because a small number were lost or a description was provided of those lost; C, follow-up rate 90% or lower (select an adequate percentage) and no description of those lost; D, no statement.

### Meta-analysis

Thirty-five observations from 33 eligible studies were pooled using a random effect model meta-analysis. We excluded the study by Jenkins et al. as an outlier due to its large standard error. The pooled effect estimate was 1.44, 95% CI (1.22–1.7), I^2^ = 70.5%. The pooled effect estimate for high quality studies was 1.39 (1.15–1.68), I^2^ = 72.3%.

**Figure 2:**
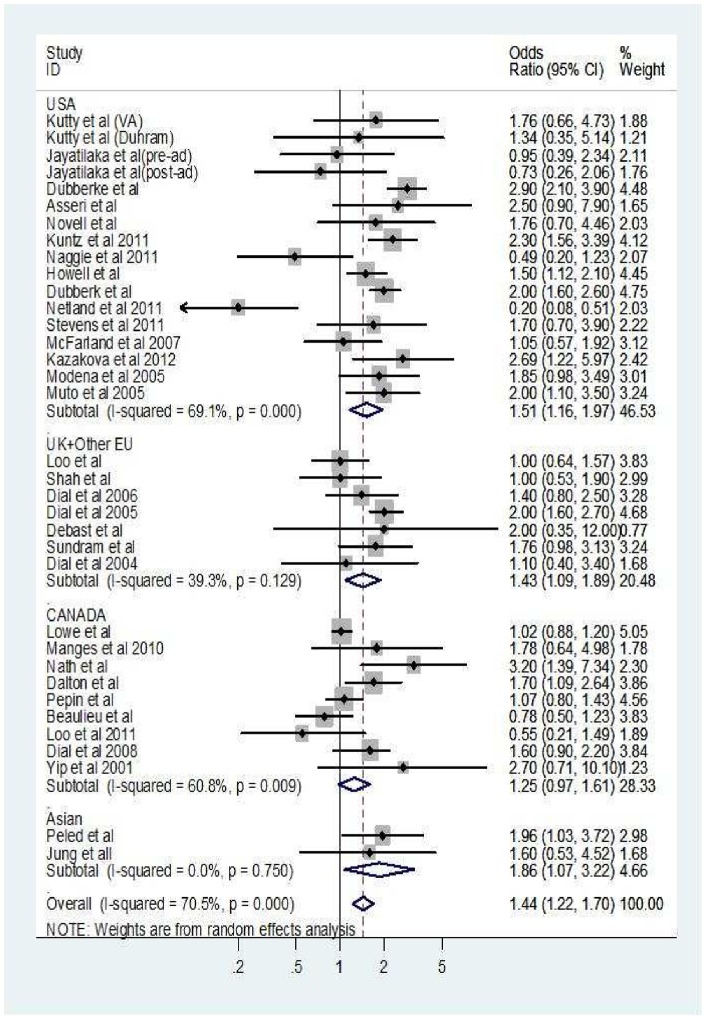
Forest plot-random effect model meta-analysis of the association between CDI and H2RAs based on 35 observations stratified by country. Error bars indicate confidence interval.

**Figure 3:**
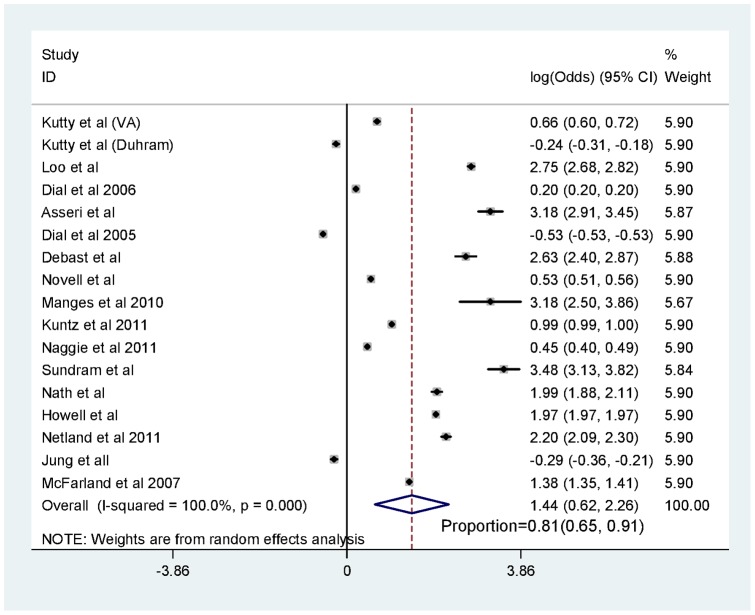
Forest plot of the pooled proportion of *Clostridium difficile* cases that were exposed to antibiotics.

Although the heterogeneity between the analyzed studies was moderate, the majority of studies pointed towards a positive association. Figure 2 shows the forest plot and the pooled effect estimate for all studies stratified by country. Table 6 summarizes the pooled estimates and associated heterogeneity across different subgroups. The pooled proportion of CDI cases that were exposed to antibiotics was 0.81, 95% CI (0.65–0.91) as shown in Figure 3.

**Table 6:** Influence of study type and country on the pooled effect estimate and its associated heterogeneity.

Group	Pooled Effect Estimate (95 % CI)	I^2^ %	Number of Observations
All studies	1.44 (1.22, 1.70)	70.5	35
Case-control studies	1.58 (1.28, 1.95)	68.9	24
Cohort studies	1.19 (0.87, 1.62)	75.6	11
Asia	1.86 (1.07, 3.22)	0	2
Canada	1.25 (0.97, 1.61)	60.8	9
Europe	1.43 (1.09, 1.89)	39.3	7
USA	1.51 (1.16, 1.95)	65.1	17

### Exploring heterogeneity

The influence of a range of a-priori selected study-level and aggregated individual-level parameters on the observed effect estimate was investigated by means of meta-regressions. Table 7 summarizes the meta-regression analyses for all 35 results. Heterogeneity could not be explained by any of the 10 considered variables.

**Table 7:** Meta-regression analysis to explore sources of heterogeneity.

	Univariate Analyses	
Study Characteristics	Coefficient	p-values
Study Design	−.27729	0.137
Low score study	.194575	0.389
Country where the study is conducted		
United States	Reference	
Canada	−.1738854	0.431
European countries	−.0849204	0.726
Asian Countries	.1809134	0.686
Setting	−.0286546	0.893
No of variables adjusted for	.0251339	0.175
Method of measuring effect estimate	−.2540725	0.325
Impact factor of the journal	−.0067289	0.380
Method of ascertainment of antibiotic		
Patient chart	Reference	
Pharmacy record	−.0139199	0.955
Interview	.3666586	0.517
Questionnaire	.2703275	0.703
Combined	.0368821	0.905
Not reported	.2469137	0.381
Proportion of antibiotic use	−.0023797	0.588

### Publication bias

**Figure 4:**
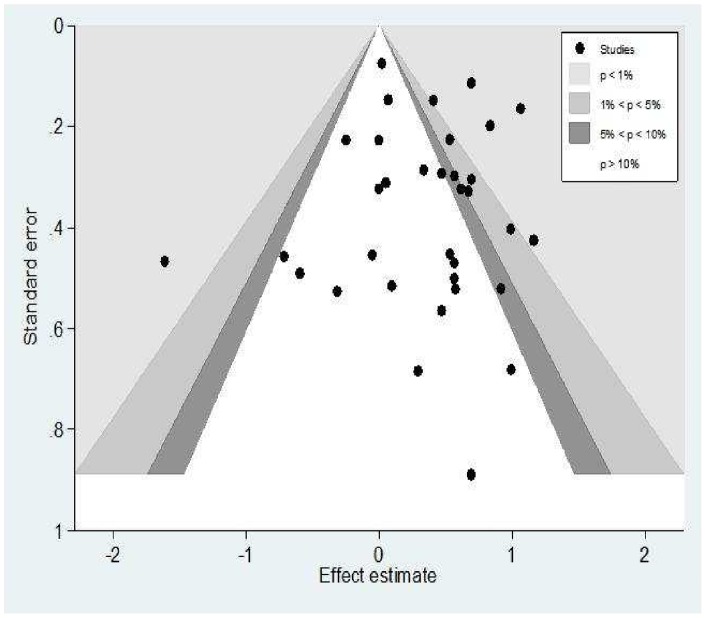
Contour-enhanced funnel plot **of the association between the estimated effect size and its standard error in all studies comparing those exposed and unexposed to H2RA displays areas of statistical significance on a funnel plot.** Contours represent conventional “milestone” levels of statistical significance (e.g., <0.01, <0.05, <0.1). This funnel plot is symmetrical as it is not missing studies in the white area excluding the possibility of publication bias (Egger's test, p = 0.905).

Figure 4 displays the contour enhanced funnel plot which showed no evidence of publication bias. This was confirmed by the Egger's test (P** = **0.905).

### Residual confounding

**Figure 5:**
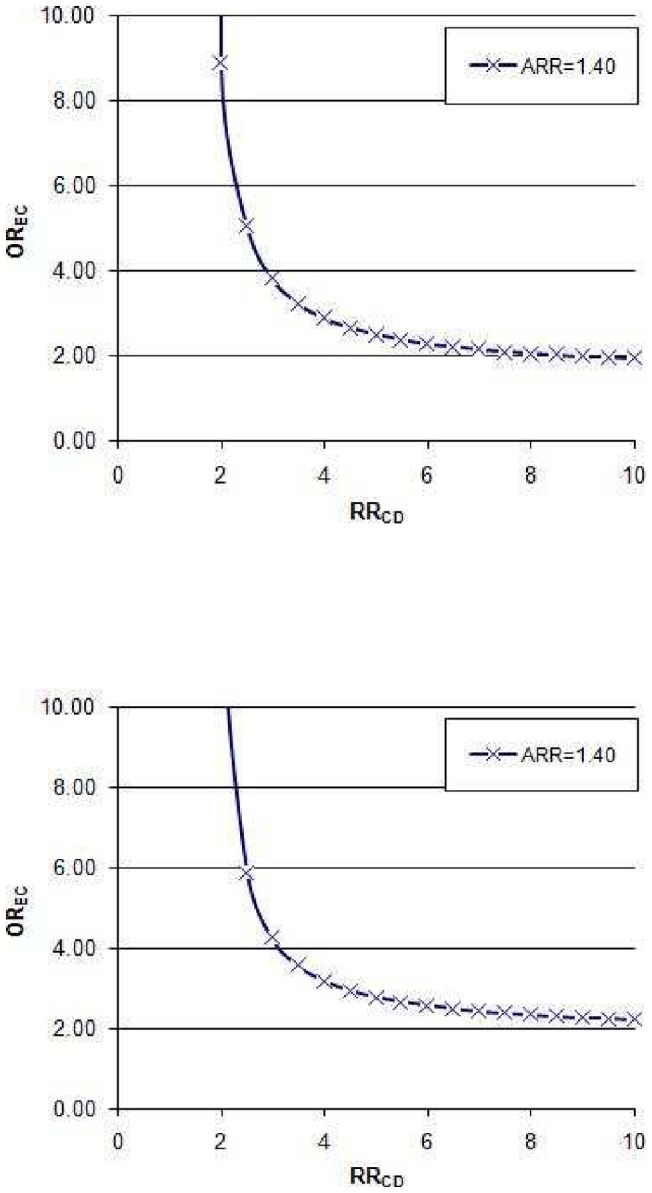
Influence of a hypothetical dichotomous confounder present in 20% (panel A) and 40% (panel B) of the study population, unaccounted for in prior adjustments performed in individual studies. The graphs depict what combinations of OREC and RR would be necessary for the confounder to fully account for the observed association between H2RA use and CDI acquisition. Abbreviations: OR_EC_, odds ratio of exposure to the confounder in H2RA non-users vs. H2RA users; RR_CD_, relative risk of CDAD in individuals exposed to the confounder vs. non-exposed.

The results of the residual confounding analysis are presented in Figure 5. Panel A refers to a confounder with a prevalence of 0.20; at this prevalence level, a strong confounder causing a two-fold increased risk of CDI would have to be severely imbalanced between H_2_ blockers users and non users (OREC  = 8.87) in order to fully account for the observed adjusted RR of 1.40. For a very common confounder with a prevalence of 0.40, stronger associations with acid-suppression use and/or CDI would be needed to explain the observed association between acid-suppression use and CDI. At this prevalence level, the confounder would have to be both imbalanced (OREC = 5.87) and increase the CDI risk (2.5-fold) to account for the observed OR, after taking publication bias into account.

### Number needed to harm

The number needed to harm (NNH) was estimated by using the pooled OR from the meta-analysis [59]. A recent large prospective hospital cohort [48] reported the incidence of CDI at 14 days after hospital admission in patients receiving antibiotics or not: which was 42/1,000 and 5.4/1000, respectively. Based on these reported baseline risks, the number needed to harm (NNH) was 58, 95% CI (37, 115) and 425, 95% CI (267, 848), respectively. For the general population, the NNH at 1 year was 4549, 95% CI (2860, 9097) at 1 year, based on a baseline incidence of CDI of 48/100,000 person-years [60].

## Discussion

### Findings

In this rigorously conducted systematic review and meta-analysis, we observed an association between H_2_RAs use and development of CDI. Using the GRADE framework, the evidence supporting this association is considered of moderate quality. Although evidence from observational studies is considered of weak quality, we have ruled out a strong effect of an unmeasured confounder and, therefore, have upgraded its quality to moderate evidence in favor of this association.

The absolute risk of CDI was highest in hospitalized patients receiving antibiotics with an estimated NNH of 58 at 2 weeks. In contrast, the risk was very low (4549) in the general population. We also observed that, on average, 19% of CDI cases had not been recently exposed to antibiotics.

These findings add to previous subgroup analyses of a limited number of H_2_RA studies performed in a recent systematic review of the association between PPI and CDI. In this review, Kwok [11] et al conducted a subgroup analysis of 15 H_2_RA studies and reported a pooled effect estimate of 1.50, 95% CI (1.23–1.83). Similarly, Leonard et al [61] reported in 2007 an analysis based on 12 studies that showed H_2_RAs use was also associated with risk of CDI with a pooled OR 1.40, 95% CI (0.85–2.29).

### Biologic plausibility

The pathogenic mechanisms operative in H_2_RAs therapy causing an increased risk of CDI acquisition are unclear, because gastric acid does not kill gastric *C. difficile* spores. One potential explanation for the association between CDI and gastric acid suppression therapies could be that the vegetative form of *C. difficile*, which is killed by acid, plays a role in pathogenesis. Vegetative forms survive on surfaces and could be ingested by patients [62]. Survival of acid-sensitive vegetative forms in the stomach could be facilitated by two primary factors: (1) suppression of gastric acid production by acid-suppressive medications; and (2) presence of bile salts in gastric contents of patients on acid-suppressive therapy. Bile salts, which are mainly found in the small intestine, are present in gastric contents, particularly among patients with gastro-esophageal reflux disease (GERD).

The extent of gastric acid suppression could play an important role in potentiating the risk of infection. Kwok [11] et at compared the risk of CDI with gastric acid suppression from 15 studies that reported on estimates of both PPI and H_2_RAs independently on their sample of participants and found that PPI is associated with higher risk of infection in comparison to H_2_RAs though both increase the risk.

### Implications

Our findings have global implications both on the inappropriate use of acid-suppression therapy and on the increasing incidence of CDI.

Given the relatively low NNH (58 patients) needed to cause a case of CDI in hospitalized patients receiving antibiotics it becomes necessary to judiciously use H_2_RAs in these patients. In addition, reducing the inappropriate use of acid-suppression medications in this patient population could lead to a significant reduction in the incidence of CDI.

On the other hand, our findings are re-assuring to the public that H_2_RAs use in the general population as over-the-counter medications do not pose significant CDI risk and is associated with a high NNH.

### Strengths

Our study has several important strengths. This review is the first systematic evaluation dedicated to examine the association between H_2_RAs and risk of CDI. It includes a comprehensive, up-to-date literature search and formal assessment of the methodological quality of pertinent studies with the largest number of relevant studies as compared to previous reviews 11,61. In addition, our pooled estimates are based on multivariate ORs of studies adjusting for several important CDI risk factors. We also performed subgroup analyses and sensitivity analyses that confirmed the robustness of our main results. There was no statistical evidence of publication bias and the effect of residual confounding on the observed association was examined. Finally, the NNH in different risk groups was calculated to aid physicians and patients in making a decision to use H_2_RA or not.

### Limitations

Our review has certain limitations. There was moderate between-study heterogeneity; however, this is often the case in meta-analyses of large observational studies [63]–[65]. Moreover the majority of studies pointed towards a positive association. There was virtually no qualitative heterogeneity, and subgroup and sensitivity analyses showed results consistent with the main analysis. There are many patient level parameters which may have led to substantial heterogeneity. Nevertheless, investigating these variables is only possible with individual patient data meta-analysis.

## Conclusions

In this rigorous systematic review and meta-analysis, we observed an association between H_2_RAs and CDI. The absolute risk of CDI associated with H_2_RAs was highest in hospitalized patients receiving antibiotics. On the other hand, our findings are re-assuring that H_2_RAs use in the general population as over-the-counter medications do not pose a significant CDI risk.

## Supporting Information

Figure S1 **PRISMA 2009 flow diagram.**
(DOC)

Table S1 **PRISMA checklist.**
(DOC)
