# Correction: The Association between Histamine 2 Receptor Antagonist Use and *Clostridium difficile* Infection: A Systematic Review and Meta-analysis

**DOI:** 10.1371/annotation/56f8945c-33f6-45bf-87ce-dd512f7c25b0

**Published:** 2013-04-02

**Authors:** Imad M. Tleyjeh, Aref A. Bin. Abdulhak, Muhammad Riaz, Musa A. Garbati, Mohamad Al-Tannir, Faisal A. Alasmari, Mushabab AlGhamdi, Abdur Rahman Khan, Patricia J. Erwin, Alex J. Sutton, Larry M. Baddour

The second author's name was spelled incorrectly. The correct name is: Aref Bin Abdulhak. 

The correct citation is: 

Tleyjeh IM, Bin Abdulhak A, Riaz M, Garbati MA, Al-Tannir M, et al. (2013) The Association between Histamine 2 Receptor Antagonist Use and Clostridium difficile Infection: A Systematic Review and Meta-analysis. PLoS ONE 8(3): e56498. doi:10.1371/journal.pone.0056498

