# Supplementary material for: The Association between Histamine 2 Receptor Antagonist Use and Clostridium difficile Infection: A Systematic Review and Meta-analysis
Source: PLoS One. 2013 Mar 4;8(3):e56498. doi: 10.1371/journal.pone.0056498 (PMC3587620; doi:10.1371/journal.pone.0056498)
Supplement: Figure S1 — PRISMA 2009 flow diagram. (DOC) [file pone.0056498.s001.doc]

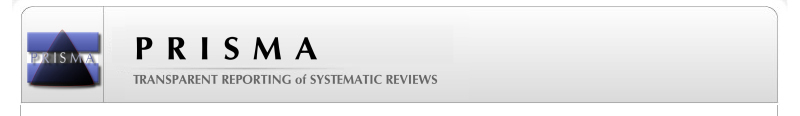
**PRISMA 2009 Flow Diagram**

**Screening**

**Included**

**Eligibility**

**Identification**

Records identified through database searching
(n =287 )

Additional records identified through other sources
(n =6 )

Records after duplicates removed
(n =?? )

Records screened
(n = 287 )

Records excluded
(n = 238 )

Full-text articles assessed for eligibility
(n = 33 )

Full-text articles excluded, with reasons
(n = 22 )

Studies included in qualitative synthesis
(n =35 )

Studies included in quantitative synthesis (meta-analysis)
(n = 35 )
