# Supplementary material for: The Association between Histamine 2 Receptor Antagonist Use and Clostridium difficile Infection: A Systematic Review and Meta-analysis
Source: PLoS One. 2013 Mar 4;8(3):e56498. doi: 10.1371/journal.pone.0056498 (PMC3587620; doi:10.1371/journal.pone.0056498)
Supplement: Table S1 — PRISMA checklist. (DOC) [file pone.0056498.s002.doc]

| **Section/topic** | **#** | **Checklist item** | **Reported on page #** |
| --- | --- | --- | --- |
| **TITLE** | | |  |
| Title | 1 | The Association Between Histamine 2 Receptor Antagonist Use and *Clostridium Difficile* Infection: A Systematic Review and Meta-analysis | 1 |
| **ABSTRACT** | | |  |
| Structured summary | 2 | **Introduction**:  *Clostridium* *difficile* infection (CDI) is a major health problem. Epidemiological evidence suggests that there is an association between acid suppression therapy and development of CDI. Purpose:  We sought to systematically review the literature that examined the association between histamine 2 receptor antagonists (H_2_RAs) and CDI.  **Methods**:  Medline, Current Contents, Embase, ISI Web of Science and Elsevier Scopus were searched for all analytical studies that examined the association between H_2_RAs and CDI from 1990 to 2012. We performed random-effect meta-analyses. We used the GRADE framework to interpret the findings.  **Results:**  Thirty-five observations from 33 eligible studies that included 201834 participants were analyzed. Studies were performed in 6 countries and nine of them were multicenter. Most studies did not specify the type or duration of H_2_RAs therapy. The pooled effect estimate was 1.44, 95% CI (1.22-1.7), I^2^= 70.5%. This association was consistent across different subgroups (by study design and country) and there was no evidence of publication bias. Meta-regression analysis of 8 study-level variables did not identify sources of heterogeneity. The number needed to harm (NNH) with H_2_RAs at 14 days after hospital admission in patients receiving antibiotics or not was 58, 95% CI (37, 115) and 425, 95% CI (267, 848), respectively. For the general population, the NNH at 1 year was 4,549 95% CI (2860, 9097).  **Conclusion**:  In this first systematic evaluation of studies that examined an association between H_2_RAs and CDI, we found that H_2_RAs use is associated with a higher risk of CDI. The absolute risk of CDI associated with H_2_RAs is highest in hospitalized patients receiving antibiotics. | 3 |
| **INTRODUCTION** | | |  |
| Rationale | 3 | *Clostridium difficile* infection (CDI) is considered a major health problem with a point prevalence of 13.1/1000 in-patient ^1^ and is increasing in incidence and mortality^2-5^. The CDI cost in the United States of America (USA) alone was conservatively estimated to exceed $1.1 billion annually^6^. Risk factors associated with CDI acquisition are numerous and traditionally have included exposure to antibiotics, advanced age, comorbidities, enteral feeding, prolonged hospitalization, endoscopy and antineoplastic medications^7-10^.  The role of gastric acid suppression therapy has gained much interest recently as a risk factor for CDI. Three recently published meta-analyses have suggested an association between gastric acid suppression therapy with proton pump inhibitors (PPI) and CDI^11-13^. The United States Food and Drug Administration (FDA) recently warned the public about a possible association between CDI and PPI use^14^.However, to date; there is no systematic review dedicated to evaluate the potential association between histamine _2_ receptors antagonists (H_2_RAs) use and risk of CDI.  H_2_RAs are popular over-the-counter (OTC) drugs worldwide^15^. Off -label use of H_2_RAs and substitution for physician care were reported in 46 % and 34% of the adult consumer, respectively^15^. Masking serious conditions, missed diagnosis, and the potential for inappropriate use by patients are concerns about OTC use of H_2_RAs^16^. Nonetheless, the implications of OTC H_2_RAs use are not yet well defined. | 5 |
| Objectives | 4 | Given the high prevalence of prescription use and OTC use of H_2_RAs and the increasing incidence and severity of CDI, we sought to systematically review the published literature that examined the association between H_2_RAs use and development of CDI following the MOOSE^17^ and PRISMA^18^ guidelines. | 5 |
| **METHODS** | | |  |
| Protocol and registration | 5 | Indicate if a review protocol exists, if and where it can be accessed (e.g., Web address), and, if available, provide registration information including registration number. | N/A |
| Eligibility criteria | 6 | Primary terms were: enterocolitis, pseudomembranous/ AND the therapeutic agents of interest:  explode omeprazole, explode proton pump inhibitors, anti-ulcer agents, and explode histamine H_2_ antagonists (Explode allows including all of the specific drugs, without having to use all of the various terms, synonyms, brands and generic names.) | 6 |
| Information sources | 7 | The initial strategy was developed in Ovid MEDLINE (1990 through January 2012), using MeSH (Medical Subject Headings) controlled vocabulary, and then modified for Ovid EMBASE (1990 through January 2012). | 6 |
| Search | 8 | Articles were limited to randomized controlled trials, cohort studies, and or case-control studies.  The same process was used with Ovid EMBASE with alterations as necessary to accommodate EMBASE's more granular subject headings.  ISI Web of Science and Elsevier Scopus use text words: (difficile OR pseudomembranous OR pseudo-membranous) AND (omeprazole OR "proton pump" OR ranitidine OR h2 OR h-2 OR "acid suppression" OR antacid*)) AND (random* OR trial* OR blind* OR cohort* OR controlled OR prospective). | 6 |
| Study selection | 9 | To be included, a study had to: (1) be an analytical study; and (2) examine the association between H_2_RAs use and incidence of CDI. | 6 |
| Data collection process | 10 | A data collection form was developed and used to retrieve information on relevant features and results of pertinent studies. Two reviewers (A.B.A. and F.A.) independently extracted and recorded data in a predefined checklist. Disagreements among reviewers were discussed with two other reviewers (I.M.T. and M.A.A.), and agreement was reached by consensus. We collected adjusted effect estimates and 95% confidence intervals (CI) based on the multivariable regression model used in each study, and the list of variables considered for inclusion in the multivariate analysis.  We used the Newcastle-Ottawa Quality Assessment Scale for cohort and case-control studies^19^which is intended to rate selection bias, comparability of the exposed and unexposed groups of each cohort, outcome assessment, and attrition bias. Two reviewers (M.A.G and F.A.) independently assessed the methodological quality of selected. Disagreement among reviewers was discussed with 2 other reviewers (I.M.T. and M.A.A.), and agreement was reached by consensus. | 7 |
| Data items | 11 | Data included the following: study characteristics (i.e., country and year of study), characteristics of the study, H2RA intake definition and ascertainment, and outcome. We also collected adjusted effect estimates and 95% confidence intervals (CI) based on the multivariable regression model used in each study, and the list of variables considered for inclusion in the multivariate analysis. | 7 |
| Risk of bias in individual studies | 12 | We used the Newcastle-Ottawa Quality Assessment Scale for cohort and case-control studies^19^which is intended to rate selection bias, comparability of the exposed and unexposed groups of each cohort, outcome assessment, and attrition bias. Two reviewers (M.A.G and F.A.) independently assessed the methodological quality of selected. Disagreement among reviewers was discussed with 2 other reviewers (I.M.T. and M.A.A.), and agreement was reached by consensus. | 9 |
| Summary measures | 13 | The primary effect measures used in the meta-analysis were Odds Ratios (OR), Hazard Ratios (HR) and Relative Risks (RR) which were assumed to reasonably estimate the same association between CDI and H2RAs because of low CDI incidence and are pooled together. Adjusted effect estimates were primarily used for this analysis. Unadjusted effect estimates were used as alternatives if studies did not pursue adjustment because of absence of association on univariate comparison. | 9 |
| Synthesis of results | 14 | We performed meta-analyses for all studies together and separately for different subgroups such as case-control studies and cohort studies.  Effect estimates from all included studies were pooled in a meta-analysis using the DerSimonian and Laird random effects model [20]. Homogeneity among studies was estimated by calculation of the variation across studies attributable to heterogeneity rather than chance (I^2^). | 9 |

Page 1 of 2

| **Section/topic** | **#** | **Checklist item** | **Reported on page #** |
| --- | --- | --- | --- |
| Risk of bias across studies | 15 | The possible influence of publication bias was graphically assessed with the novel method of contour-enhanced funnel plot where log-transformed odds ratios were plotted against standard errors. This method examines whether any funnel plot asymmetry is likely to be due to publication bias compared with other underlying causes of funnel plot asymmetry. The contours help to indicate whether areas of the plot, where studies are perceived to be missing, are where studies would have statistically significant effect sizes or not and thus decrease or increase the evidence that the asymmetry is due to publication bias. The presence of funnel plot asymmetry was also assessed using Egger's test.^21^ | 9 |
| Additional analyses | 16 | Finally, the possible influence of unknown confounders (residual confounding) was investigated with a rule-out approach described by Schneeweiss^22^. This approach stipulates the influence of a hypothetical confounder and determines what characteristics this confounder must have to fully account for the observed association between use of H_2_RAs and occurrence of CDI. The hypothetical confounder is characterized by its association to H_2_RAs use (OR_EC_, odds ratio of exposure to the confounder) and its association to the outcome (RR_CO_, relative risk of outcome in individuals exposed to the confounder vs. non-exposed). For this analysis, the absolute risk in the pooled non-exposed group was used for conversion of odds ratio to relative risk using the method described by Zhang and Yu.^23^ Separate analyses were performed to demonstrate what levels of OR_EC_ and RR_CO_ would be required to fully explain the observed association between H_2_RAs and CDI for different hypothetical prevalence of the unknown confounder (i.e. P_C_=0.2, P_C_=0.4) before and after adjustment for publication bias as described above.  In all analyses, results associated with p-values <0.05 (two-sided test) were considered statistically significant. All statistical analyses were performed using Stata version12 statistical software (StataCorp, College Station, Texas) | 9 |
| **RESULTS** | | |  |
| Study selection | 17 | 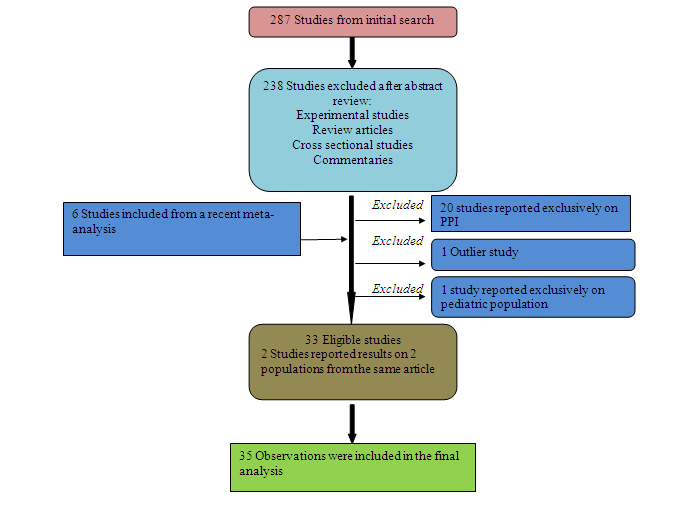 | 34 |
| Study characteristics | 18 | The search yielded 27 eligible studies after excluding 260 citations. Six more studies were retrieved from recent review articles and added to the total eligible studies. Kutty^24^ et al and Jayatilaka^25^ et al, each reported 2 different observations for different participants. Thus, a total of 33 articles met our inclusion criteria representing 35 Observations that included 201834 participants. The study selection process is illustrated in Figure 1 and the main characteristics of the included studies are summarized in Table 1. Twenty-four case control studies^24-34,42,43,47-49,51-56^ and 11 cohort studies^35-41,44-46,50^ reported data on both community-acquired and hospital-acquired CDI (8 observations were from community-acquired CDI, 23 from hospital-acquired and 4 representing both type of CDI). Six studies^24,36,46,49,51,52^ were from multiple centers; two from UK general practice research database^26,28^, and the remaining were from single centers. The included studies were performed in 6 countries (17 studies from USA, 9 from Canada, 6 from United Kingdom, 1 from Netherlands, 1 from Israel, and one from Korea). Most studies did not specify the type or duration of therapy with H2RAs. Table 2 and 3 summarized the case ascertainment, control or non-exposed group selection method for case control and cohort studies, respectively. | 10,11 |
| Risk of bias within studies | 19 | Quality assessment of all included studies was done using the validated Newcastle-Ottawa Quality Assessment Scale^19^ for cohort and case control studies (Table 4 and 5). Most studies were of good quality with no evidence of selection bias, and with good comparability of the exposed and unexposed groups of each cohort, and outcome assessment. | 12 |
| Results of individual studies | 20 | 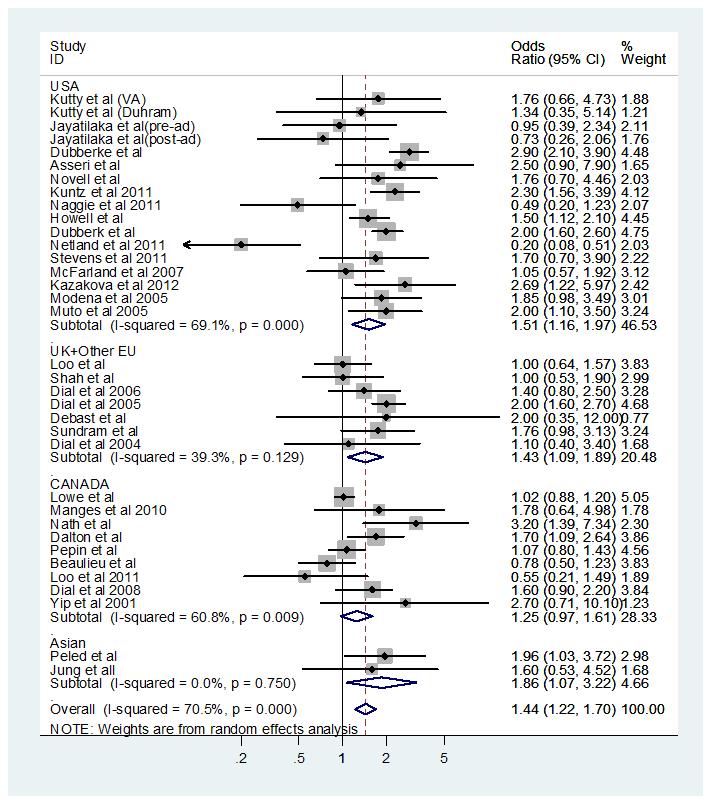 | 35 |
| Synthesis of results | 21 | Thirty-five observations from 33 eligible studies were pooled using a random effect model meta-analysis. We excluded the study by Jenkins et al. as an outlier due to its large standard error. The pooled effect estimate was 1.44 (1.22-1.7), I^2^= 70.5%. Although the heterogeneity between the analyzed studies was high, the majority of studies pointed towards a positive association. Figure 2 shows the forest plot and the pooled effect estimate for all studies stratified by country. Table 6 summarizes the pooled estimates and associated heterogeneity across different subgroups. Pooled proportion of CDI cases that were exposed to antibiotics from 17 studies were 0.81, 95% CI (0.65-0.91)  Exploring heterogeneity:  The influence of a range of a-priori selected study-level and aggregated individual-level parameters on the observed effect estimate was investigated by means of meta-regressions. Table 7 summarizes the meta-regression analyses for all 35 results. Heterogeneity could not be explained by any of the 8 considered variables. | 11,12 |
| Risk of bias across studies | 22 | Figure 3 displays the contour enhanced funnel plot which showed no evidence of publication bias. This was confirmed by the Egger's test (P**= 0.905).** | 12 |
| Additional analysis | 23 | Separate analyses were performed to demonstrate what levels of OR_EC_ and RR_CO_ would be required to fully explain the observed association between H_2_RAs and CDI for different hypothetical prevalence of the unknown confounder (i.e. P_C_=0.2, P_C_=0.4) before and after adjustment for publication bias as described above. | 12 |
| **DISCUSSION** | | |  |
| Summary of evidence | 24 | In this rigorously conducted systematic review and meta-analysis, we observed an association between H_2_RAs use and development of CDI. The absolute risk of CDI was highest in hospitalized patients receiving antibiotics with an estimated NNH of 58 at one year. In contrast, the risk was very low (4,549) in the general population. We also observed that, on average, 19% of CDI cases had not been recently exposed to antibiotics.  Although the pooled effect estimate is based on data adjusted for potential confounders, such as differences in age, sex, antibiotic intake and co-morbid conditions; and because all included studies were of observational design and patients were not randomly assigned to H_2_RAs therapy, we calculated the magnitude of association with H_2_RAs use and development of CDI that a hypothetical confounder would need to fully account for the apparent H_2_RAs effect. This analysis showed that only a very strong confounder would be able to explain the association seen in the main analysis. Hence, it seems unlikely that the observed association is entirely due to confounding and that H_2_RA is completely devoid of a causal influence on CDI. In addition, it should be noted that the confounding model employed stipulates a confounder entirely independent of factors adjusted for in individual studies. If, as is often the case, the unknown confounder is correlated to confounders already adjusted for, its impact could be much smaller than the model indicates. | 13 |
| Limitations | 25 | Our review has certain limitation. There was significant between-study heterogeneity; however, this is often the case in meta-analyses of large observational studies^61-63^, the majority of studies pointed towards a positive association. There was virtually no qualitative heterogeneity, and subgroup and sensitivity analyses confirmed robustness by showing results similar to the main analysis. Finally, we did not have patient-level data, the gold standard method to test for interactions at the patient-level covariates. | 16 |
| Conclusions | 26 | This is the first systematic evaluation of studies that examined an association between H_2_RAs and CDI and the results suggested that H_2_RAs use was associated with a higher risk of CDI. The absolute risk of CDI associated with H_2_RAs was highest in hospitalized patients receiving antibiotics. On the other hand, our findings are re-assuring that H_2_RAs use in the general population as over-the-counter medications do not pose a significant CDI risk. | 16 |
| **FUNDING** | | |  |
| Funding | 27 | No funding was received for the production of this work | 17 |

*From:*  Moher D, Liberati A, Tetzlaff J, Altman DG, The PRISMA Group (2009). Preferred Reporting Items for Systematic Reviews and Meta-Analyses: The PRISMA Statement. PLoS Med 6(6): e1000097. doi:10.1371/journal.pmed1000097

For more information, visit: **www.prisma-statement.org**.

Page 2 of 2
